# Quantitative Genetic Mapping and Genome Assembly in the Lesser Wax Moth *Achroia grisella*

**DOI:** 10.1534/g3.119.400090

**Published:** 2019-05-17

**Authors:** Boryana S. Koseva, Jennifer L. Hackett, Yihong Zhou, Bethany R. Harris, John K. Kelly, Michael D. Greenfield, Jennifer M. Gleason, Stuart J. Macdonald

**Affiliations:** * K-INBRE Bioinformatics Core, University of Kansas, Lawrence, Kansas 66045; † Department of Ecology and Evolutionary Biology, University of Kansas, Lawrence, Kansas 66045; ‡ Department of Molecular Biosciences, University of Kansas, Lawrence, Kansas 66045; § Equipe Neuro-Ethologie Sensorielle, ENES/Neuro-PSI, CNRS UMR 9197, Université de Lyon/Saint-Etienne, 42023 Saint Etienne, France; ** Center for Computational Biology, University of Kansas, Lawrence, Kansas 66047

**Keywords:** QTL, male song, genome assembly, genotyping-by-sequencing

## Abstract

Specific characteristics of the male *Achroia grisella* acoustic mating signal determine a male’s attractiveness toward females. These features are genetically variable in populations, and mapping experiments have been used to identify loci contributing to song variation, and understand the evolutionary forces acting on this important sexual trait. Here we built on this foundation and carried out QTL (Quantitative Trait Locus) mapping using >1,000 recombinant individuals, genotyping this large cohort at thousands of sequence-based markers covering the entire collection of 30 *A. grisella* chromosomes. This dense marker set, coupled with our development of an annotated, draft genome of *A. grisella*, allowed us to link >3,000 genome scaffolds, >10,000 predicted genes, and close to 275Mb of genome sequence to chromosomes. Our QTL mapping confirmed a fraction of the QTL identified in a previous study, and additionally revealed novel loci. Collectively, QTL explained only small fractions of the phenotypic variance, suggesting many more causative factors remain below the detection threshold of our study. A surprising, and ultimately challenging feature of our study was the low level of intrachromosomal recombination present in our mapping population. This led to difficulty ordering markers along linkage groups, necessitating a chromosome-by-chromosome mapping approach, rather than true interval mapping, and precluded confident ordering/orienting of scaffolds along each chromosome. Nonetheless, our study increased the genomic resources available for the *A. grisella* system. Enabled by ever more powerful technologies, future investigators will be able to leverage our data to provide more detailed genetic dissection of male song variation in *A. grisella*.

The advent of relatively inexpensive, high-throughput sequencing technologies has paved the way for sophisticated genetic, genomic, and evolutionary analysis in organisms outside of humans and the traditional model systems (Ellegren 2014). Indeed, such approaches have enabled the assembly and annotation of genomes for an increasingly large number of invertebrates, such as the non-model Drosophilid *Drosophila suzukii*, an invasive pest species (Chiu *et al.* 2013), the clam shrimp *Eulimnadia texana*, which has a unique sex-determination system (Baldwin-Brown *et al.* 2018), and the orb-weaving spider *Nephila clavipes*, which generates several different silks with diverse properties (Babb *et al.* 2017). In concert, several major community efforts are underway to sequence thousands of invertebrate and vertebrate species (Genome 10K Community of Scientists 2009; Robinson *et al.* 2011; i5K Consortium 2013; Koepfli *et al.* 2015; Zhang *et al.* 2015; Jarvis 2016). Sequencing technologies are also being used to characterize genomewide variation among individuals/strains via genotyping-by-sequencing (Baird *et al.* 2008; Andolfatto *et al.* 2011; Elshire *et al.* 2011), and such data allow a range of population genomic and phylogenomic questions to be addressed. For instance, these approaches have been used to examine genomic signatures of local adaptation during invasion to new habitats in the monkeyflower *Mimulus guttatus* (Monnahan *et al.* 2015), and to understand the diversification of snake species in the *Boa* complex (Card *et al.* 2016). Genotyping-by-sequencing has also been successful in building genetic linkage maps (*e.g.*, Koseva *et al.* 2017; Nie *et al.* 2017) improving on maps generated using anonymous AFLP markers or small numbers of sequence-based variants (*e.g.*, microsatellites). Merging genome assemblies with linkage maps constructed using large numbers of sequence-based markers can facilitate the dissection of complex traits in any organism.

We leverage advances in sequencing to explore genetic variation for a complex reproductive behavior exhibited by the lesser wax moth, *Achroia grisella* (Lepidoptera: Pyralidae), a honeybee symbiont. In most moth species females attract males by emitting long-distance advertisement pheromones (Greenfield 1981), and in some species the male produces a courtship sound when he arrives in the vicinity of the female. In contrast, *A. grisella* males remains stationary and attract females over a long distance largely via an intense ultrasonic advertisement song (Greenfield and Coffelt 1983). While intense sound production is found in various moth species, such sounds are normally produced by both sexes, during flight, and in the context of interacting with, and deterring predatory bats (Conner and Corcoran 2012; Barber and Kawahara 2013). Although *A. grisella* males and females do hear and avoid echolocating bats (Alem *et al.* 2011), no evidence indicates that the male *A. grisella* courtship song functions as a bat deterrent.

Male *A. grisella* generate their sounds using a pair of organs called tymbals located at the base of each forewing (Spangler *et al.* 1984). Fanning of the wings causes the tymbals to buckle both during the upward and downward wing strokes, in each case generating a pulse of high-frequency sound (Spangler *et al.* 1984). Despite an additional pheromonal release by the male, playback experiments using a loudspeaker have demonstrated that the male call alone is sufficient to attract female *A. grisella* (Spangler *et al.* 1984; Jang and Greenfield 1996). Male songs can vary in the loudness, or amplitude of the acoustic signal, in the rate with which the song is produced - termed the pulse pair rate, and in the time between the paired pulses - termed the asynchrony interval (Jang and Greenfield 1996). A series of playback experiments employing synthetic songs have shown that females prefer signals that are louder (*i.e.*, have higher amplitude), that are delivered at a faster pulse pair rate, and that include longer intervals between the pairs of pulses (Jang and Greenfield 1996, 1998; Limousin and Greenfield 2009).

Each song trait exhibits significant additive genetic variance (Collins *et al.* 1999; Brandt and Greenfield 2004), and three previous studies have genetically dissected this variation, mapped QTL (Quantitative Trait Loci), and gained insight into the selective forces acting on male song (Limousin *et al.* 2012; Alem *et al.* 2013; Gleason *et al.* 2016). However, the limited genetic toolbox of *A. grisella* hindered these efforts. While all three prior mapping studies generated a genetic linkage map on which to place mapped QTL, in two cases (Limousin *et al.* 2012; Alem *et al.* 2013) the maps were constructed with a genomewide set of AFLP markers, and in the other case (Gleason *et al.* 2016) the map is based on a modest number of EST-based markers. Thus, many QTL for male song traits are mapped to anonymous locations, and cannot be compared across studies, and some true, causative loci may have been missed due to a failure to tag all chromosomes. In addition, without a genome reference underlying the genetic map, one can only speculate about the genes involved.

In this work we expand upon the mapping study reported by Gleason *et al.* (2016), examining a larger pool of phenotyped animals derived from the same mapping cross. We employ a genomewide set of markers derived from genotyping-by-sequencing, and create a sequence-based genetic linkage map. We also build a draft, *de novo* assembly of the *A. grisella* genome with a set of robust gene annotations, and tie the annotated genome scaffolds to the linkage map. Finally, we identify additional QTL not identified in the original study by Gleason *et al.* (2016), and provide intriguing evidence supporting a very low crossover rate in the mapping population of *A. grisella* used in our study.

## Materials and Methods

### QTL mapping populations

Mapping populations were derived by intercrossing inbred strains derived from individuals collected in Kansas (KS) and in Florida (FL), as described in Gleason *et al.* (2016). KS strain females were crossed to FL strain males to generate F_1_ progeny, and F_1_ animals were backcrossed individually to either the KS or FL strain to generate a series of experimental families. The males of these families were phenotyped and genotyped for QTL mapping. Because *A. grisella* females show no germline meiotic crossing over (Suomalainen *et al.* 1973), to generate recombinant individuals, F_1_ males were backcrossed to KS females (hereafter “Kansas Backcross” or “KS-BC”) or to FL females (hereafter “Florida Backcross” or “FL-BC”). In addition, F_1_ females were crossed to KS males to produce a population of segregant individuals (hereafter “Kansas Segregants” or “KS-SG”).

### Phenotypes

All experimental backcross and segregant males were phenotyped for three song traits that influence male attractiveness to females (pulse-pair rate, asynchrony interval, and peak amplitude), along with development time and body weight. Pulse-pair rate is the rate (in msec) at which the male song pulses are emitted. Pulses are normally produced in left-right pairs, and asynchrony interval is the time (in μsec) between these paired pulses. Peak amplitude is the highest sound pressure level recorded during a pulse. Development time is the number of days between oviposition and adult eclosion, and body weight (in mg) is measured immediately following eclosion. Full details of the phenotyping is described by Gleason *et al.* (2016). Recombinant males were reared and phenotyped across 2 years, with around half of the experimental families being produced in 2007 and half in 2008. All segregants were phenotyped in a single year.

### Genotyping-by-sequencing library construction

We isolated DNA from 17 KS individuals, 14 FL individuals, 5 F_1_ offspring, 447 KS-BC recombinants, 465 FL-BC recombinants, and 198 KS-SG segregants. The DNA was then used to generate a multiplexed genotyping-by-sequencing library using a modified MSG protocol (Andolfatto *et al.* 2011). We made two modifications. First, we employed the restriction enzyme *AseI*, rather than *MseI*, as the former was expected to cut less frequently, and lead to higher read counts at marker sites. Second, we used a set of 48, 6-mer in-line barcodes (corresponding to the first 6-bp of the Illumina Read1 sequence) in combination with a set of 24 i7 index sequences added during PCR, to allow all of our test samples to be multiplexed and sequenced together. The multiplexed pool was sequenced over multiple lanes of an Illumina HiSeq2500 instrument (KU Genome Sequencing Core) generating 100-bp single-end reads.

### Marker discovery

For genotyping-by-sequencing analysis we made use of the Stacks pipeline (Catchen *et al.* 2011). We demultiplexed reads with the *process_radtags* algorithm, and merged all reads associated with the KS and FL parental individuals into two pools (KS-pool = 16.3 million reads, FL-pool = 13.8 million reads). We then used *ustacks* to *de novo* assemble loci for each parental pooled sample. We parameterized *ustacks* to construct stacks with a minimum coverage (*m*) of 5, a maximum number of differences between reads within a locus (*M*) of 2, no secondary alignments (*N* = 0), a maximum number of stacks per *de novo* locus (*max_locus_stacks*) equal to 2, and with the “Removal” and “Deleveraging” algorithms enabled (−*r* and –*d*, respectively). The Removal algorithm excludes stacks that are highly repetitive, while the deleveraging algorithm attempts to resolve over-merged stacks. In total, 148,448 and 143,201 stacks were assembled for the KS and FL parental strains, respectively. We then used *cstacks* to merge loci from the two parental pooled samples. We set the maximum distance allowed between loci (*n*) to 2, allowing for the alleles at any heterozygous loci in the parental strains to be merged. We interrogated this catalog and identified 72,076 entries in which each parent contributed a single monomorphic allele. We excluded all other loci because they could represent heterozygous loci, alleles sampled in only one of the two parental lines, or incorrectly-merged paralogous sequences. Of the 72,076 loci, 26,905 (37.3%) were polymorphic between the FL and KS parental lines, and represent the set of informative markers used to genotype the recombinant and segregant progeny.

### Marker genotyping

To call genotypes in the recombinant and segregant populations, we first *de novo* assembled loci for each individual using *ustacks* with similar parameterization to that used for the KS and FL parental samples described above (*m* = 2, *N* = 2, –*r*, –*d*, –*max_locus_stacks* = 2). The loci for each individual were then matched against the catalog of informative loci generated using only the parental lines. Finally, we used the *genotypes* routine within Stacks to generate genotype calls for all recombinants and segregants. Genotyping rates in the three mapping populations were 49.5% (KS-BC), 42.0% (FL-BC), and 34.2% (KS-SG).

### Assigning markers to linkage groups

To localize markers we used Lep-MAP2 (Rastas *et al.* 2013, 2016), first converting the Stacks *genotypes* output file - containing progeny and parents - to the required LINKAGE pedigree format (Lathrop *et al.* 1984). Within Lep-MAP2 we first used the *Filtering* module to remove loci showing segregation distortion (*dataTolerance* = 0.01) in the two recombinant backcrosses, and to remove loci/individuals with limited genotyping data. Different filtering criteria were employed for each backcross to maintain similar levels of missing data across the three sets of genotypes. For the KS-BC we retained 8,132 markers and 339 individuals, for the FL-BC we retained 5,970 markers and 313 individuals, and for the KS-SG we retained 13,295 markers and 198 individuals.

We assigned markers to linkage groups independently for each of the three mapping populations using the *SeparateChromosomes* Lep-MAP2 module, requiring a minimum LOD (logarithm of odds) score of 20, and a minimum of 20 markers per linkage group. To establish linkage group homology across populations we compared the assignment of markers to linkage groups in each map. Eight (0.2%) of the 3,204 overlapping markers between KS-BC and FL-BC were removed entirely from the dataset because they were inconsistent in their placement, *i.e.*, a marker was placed on different linkage groups in the two maps. Based on the remaining overlapping markers, linkage groups in KS-BC and FL-BC were renamed to reflect the respective linkage groups in KS-SG.

For the pair of recombinant backcross populations, markers associated with each linkage group were then separately ordered with *OrderMarkers* using the Kosambi function (*useKosambi* = 1; Kosambi 1943), taking into account the achiasmatic meiosis in females (*initRecombination* = 0.05 0, where the first number is the *a priori* probability of crossing over in males, and the second is the same probability in females), and removing markers positioned at identical genetic positions within each linkage group (*removeDuplicates* = 1). Following this procedure, we immediately noticed that marker order within a given linkage group was very different between backcrosses, particularly for markers in the middle of each linkage group. We examined this observation in multiple ways (see “Results and Discussion”), concluding there are remarkably few crossover events evident the data, precluding confident marker ordering within linkage groups.

### QTL mapping

Due to the limited number of crossovers, and the inability to order markers, we elected not to attempt traditional interval QTL mapping (Lander and Botstein 1989) for the backcross populations. Instead, we ignored all crossing over, and assigned a single consensus genotype to each linkage group in each individual based on three filters: (1) at least 80% of the markers on the linkage group were given a genotype call, (2) the minimum number of called markers for that linkage group was 30, and (3) at least 90% of the markers on that linkage group had the same genotype. We used these “collapsed” genotypes to perform marker regression in R/qtl (Broman and Sen 2009), effectively mapping QTL to the chromosome level for each phenotype. Given the family structure of the mapping populations, and given this structure is confounded with the year in which the experimental individuals were generated, we included family membership as a covariate in all QTL analyses. We additionally performed QTL mapping for both pulse-pair rate and peak amplitude after correcting for body weight variation (see “Results and Discussion”). Within each mapping population we regressed weight on each of these song traits individually using the *glm* function in R (R Core Team 2018), extracted the residuals, and used these weight-corrected phenotypes for mapping. Significance thresholds for QTL mapping were established by running 1,000 permutations of the data (Churchill and Doerge 1994).

We used R/qtlDesign (Sen *et al.* 2007) to calculate the statistical power we had to detect each of the QTL mapped in the study. We used the function *powercalc* for each QTL we detected with the following parameters: (1) sample size, (2) error variance, as calculated via the R/qtl *fitqtl* function for each QTL using the Haley-Knott regression method, and (3) the QTL effect, also calculated via *fitqtl*. The cross type was set to ‘bc’ for all power calculations.

### Genome sequencing data collection

DNA was isolated from multiple males from the KS *A. grisella* inbred line (Gleason *et al.* 2016) and pooled. We employed males, the homogametic (ZZ) sex, to ensure that the Z had similar coverage to each autosome in subsequent sequencing. Two Illumina sequencing libraries were generated (Cofactor Genomics, Inc.); a short insert size paired end (hereafter “PE”) library with 280-504bp inserts, and a long insert size mate pair (hereafter “MP”) library with 3-5kb inserts. Each library was sequenced on three lanes of an Illumina HiSeq2500 instrument, one lane at Cofactor Genomics, Inc. and two lanes at the KU Genome Sequencing Core. We obtained 291 million and 405 million 101-bp read pairs for the PE and MP library, respectively (Table S1).

### Genome sequencing data processing

Read quality has a major effect on the result of *de novo* genome assembly (Salzberg *et al.* 2012), therefore we first preprocessed the raw FASTQ files. Initially, we removed adaptor sequences using Scythe (https://github.com/vsbuffalo/scythe) and quality trimmed reads via Sickle (https://github.com/najoshi/sickle), eliminating any reads containing uncalled positions (*i.e.*, “N” bases), or with a trimmed length below 80-bp. Any reads whose pairs were discarded in the trimming process were saved in a separate FASTQ file which was used alongside the pairs in downstream analyses. Subsequently, we used bowtie2 (Langmead and Salzberg 2012) with default settings to align preprocessed reads to the PhiX (*Escherichia* virus phiX174) reference genome (NCBI Reference Sequence: NC_001422.1) that was run together with our sample as a control during Illumina sequencing, removing any contaminating reads, and storing the unmapped reads. Finally, we corrected the set of uncontaminated, quality-trimmed reads using Quake, a maximum-likelihood based tool for detecting and correcting sequencing errors (Kelley *et al.* 2010), setting *k* to 18. Overall, preprocessing removed 10% of the original reads (Table S1), slightly improving the average base quality (Figure S1).

### Genome assembly and evaluation

To assemble processed reads into scaffolds we used the de Bruijn assembler ABySS (Simpson *et al.* 2009), selecting this software both due to its low error rate when assembling a human chromosome (Salzberg *et al.* 2012), and its parallel processing ability and low memory requirements (Simpson *et al.* 2009). ABySS uses PE reads to assemble contigs, and then MP reads to construct scaffolds. In common with most de Bruijn assemblers (Pevzner *et al.* 2001) ABySS requires that the user specify a *k*-mer size, where the optimal *k* depends on the repetitiveness of the genome, its heterozygosity, and technology-specific error rates (Chikhi and Medvedev 2014). We used KmerGenie (Chikhi and Medvedev 2014) to estimate the appropriate *k*-mer as 93, then ran ABySS with this value, otherwise employing the default parameters, on a single cluster node with 16 processors and 32GB of RAM. To assess completeness of the final draft assembly we used CEGMA (Parra *et al.* 2007, 2009) with default parameters.

### RNAseq and transcriptome assembly

To assist with genome annotation, we collected RNAseq data and assembled transcripts from *A. grisella*. Total RNA was individually isolated from two pupae from strain “Louisiana line 112” (Zhou *et al.* 2008). A poly-A selected, unstranded TruSeq Illumina RNAseq library was constructed for each individual, and the pair of libraries were sequenced over a single lane of a HiSeq2500 instrument to generate around 175 million paired-end 100-bp reads (KU Genome Sequencing Core). Quality trimming via Sickle (https://github.com/najoshi/sickle, window-wise quality threshold parameter *q* = 40, minimum read length post-trimming = 50-bp) retained around 96% of the reads. All quality-trimmed reads were merged into a single FASTA file. To assemble transcripts we used Trinity (Grabherr *et al.* 2011) with default parameters, except we turned on read normalization, and set the maximum read coverage for normalization to 50. The assembly took <6 hr on a 16-core node with 256 GB of RAM, and generated 96,420 transcripts with an N50 of 2551-bp and a mean scaffold length of 1178.45 bp.

### Genome annotation

We annotated our *de novo* genome assembly using MAKER2 (Holt and Yandell 2011). We provided MAKER2 with the Trinity-assembled *A. grisella* transcripts, protein databases from the lepidopterans *Heliconius melpomene* (Davey *et al.* 2016), *Danaus plexippus* (OGS2.0; http://monarchbase.umassmed.edu/), and *Bombyx mori* (Xia *et al.* 2004), and a repeat database generated by RepeatModeler (Smit and Hubley 2008-2015) that contains short and long interspersed nuclear elements, long terminal repeat elements, small RNAs, and other unclassified repeats. Within MAKER2 we used two gene predictors, Augustus (Stanke and Morgenstern 2005) and SNAP (Korf 2004). For Augustus, we employed a publicly-available parameter set developed for *H. melpomene*, which is distributed with the software. For SNAP, we used an HMM file generated by bootstrap training of the gene predictor over three runs of MAKER2. Assessment of the completeness of the genome annotation was accomplished using BUSCO (version 3, Simão *et al.* 2015) using single-copy orthologs specific to the phylum Arthropoda from OrthoDB (version 9, Waterhouse *et al.* 2013) as our reference gene set.

### Associating genome scaffolds with linkage groups

We created a nucleotide database from the genome assembly using *makeblastdb* on a local BLAST (Altschul *et al.* 1990) installation. The sequences of markers that had been placed on linkage groups were then extracted from the Stacks *cstacks* catalog file, and formatted as a FASTA file, adding the linkage group for each marker to the sequence header. Each marker sequence was then mapped to the assembled genome using BLASTN (−*evalue* 1e-30), and 9,746 markers hit just one scaffold (59 markers with significant hits to more than one scaffold were ignored), enabling us to place scaffolds onto linkage groups. Similarly, we placed the sequences of the 75 EST-derived markers used by Gleason *et al.* (2016) onto scaffolds. This allowed us to translate among the linkage group identifiers assigned in our two studies (Table S2).

### Comparing genotypes across studies

In their QTL mapping study Gleason *et al.* (2016) employed some of the same recombinant individuals we used here, genotyping a set of markers using Illumina BeadXpress technology. Because we know the scaffolds on which all markers from this and the present study reside (above), we could compare the accuracy of genotype calls at pairs of markers on the same scaffold. We note that the assumption such markers should have the same genotype is dependent on scaffolds rarely being chimeric, and on crossovers being infrequent at the scale of a given scaffold. We examined only those 32 Gleason *et al.* (2016) markers present on scaffolds associated with the 16 linkage groups showing an unambiguous one-to-one relationship among studies (Table S2), minimizing the potential for marker-to-scaffold mismapping to falsely indicate an apparently high rate of genotyping error. When multiple markers from the present study were on one of these scaffolds we assigned a consensus genotype call for each individual, assigning a no-call in the case that marker-specific calls were not all identical.

### Associating A. grisella linkage groups with chromosomes from sequenced lepidopterans

The genome of *B. mori* (version Jan. 2017) was downloaded from SilkBase (http://silkbase.ab.a.u-tokyo.ac.jp/cgi-bin/index.cgi), and the genome of *H. melpomene* (version 2.5), along with the accompanying AGP file (A Golden Path file, which is a description of the assembly), was downloaded from the Butterfly Genome Database (http://butterflygenome.org/). We generated a BLASTable database for each genome, and subsequently mapped repeat-masked *A. grisella* scaffolds (with repetitive sequence replaced by runs of “N” bases) against each database using BLAST+ with default parameters (Camacho *et al.* 2009). We ignored alignments based on the following criteria: (a) alignments of *A. grisella* scaffolds not assigned to linkage groups, (b) alignments to *B. mori* sequences not assigned to chromosomes, (c) alignments with an identity percentage of <90%, and (d) alignments <125-bp long (for *B. mori*) or <100-bp long (for *H. melpomene*). The remaining alignments were considered, and an *A. grisella* linkage group was associated with a chromosome from one of the other species if at least 15 alignments were identified.

### Independent identification of the Z chromosome

To identify the Z using an orthogonal approach, we extracted protein sequences of Z-linked genes from the two lepidopteran species *B. mori* (Xia *et al.* 2004) and *Melitaea cinxia* (Ahola *et al.* 2014). Each set of protein sequences was aligned to our assembled genome using TBLASTN (−*evalue* 1e-50). Proteins mapping to a single scaffold were used to identify a set of *A. grisella* scaffolds containing sequences with strong homology to known Z-linked proteins. Since scaffolds are associated with linkage groups (see above), we confirmed the identity of the linkage group representing the *A. grisella* Z chromosome.

### Data availability

We have deposited raw FASTQ files from our genome sequencing, RNAseq, and MSG in the NCBI Sequence Read Archive under accession SRP158931. In addition, we have deposited a data package on FigShare that contains the following information, resources and scripts: (1) all supplementary tables and figures described in the text, (2) phenotypes for all individuals, (3) genotypes for all markers associated with linkage groups, (4) genotypes for the consensus, linkage group-specific markers used for QTL mapping, (5) sequences of all markers, all assembled genome scaffolds, and all assembled transcripts, (6) the genome annotation, and (7) custom Python scripts employed. Supplemental material available at FigShare: https://doi.org/10.25387/g3.8072405.

## Results and Discussion

This study develops the previous genetic mapping of male song and life history characters in the lesser wax moth *Achroia grisella*, expanding on the sample of recombinant individuals originally described by Gleason *et al.* (2016). We employed around 1,000 phenotyped males from three mapping populations, each established from the same pair of parental strains, used genotyping-by-sequencing to generate markers and resolve linkage groups for the full complement of chromosomes, and subsequently identified several loci contributing to trait variation. In addition, by generating a draft, annotated *de novo* genome assembly for *A. grisella*, and linking large numbers of scaffolds to linkage groups, we facilitate continued genetic analysis of male song in this non-model insect species.

### Phenotypic variation

Three male song traits (pulse-pair rate, peak amplitude, asynchrony interval) and two life history traits (development time and body weight) were measured in two recombinant backcross populations (KS-BC and FL-BC) and in one segregant population (KS-SG). All traits exhibited substantial variation within populations (Table 1), as was previously observed by Gleason *et al.* (2016). Using simple linear models we found that “family” influenced development time in all three populations, body weight in KS-BC and KS-SG, and peak amplitude in KS-BC (Table 1; *P* < 0.001 in each case).

**Table 1:** Phenotype means for each family and mapping population

Popn	Year*^a^*	Family ID*^a^*	N	Development time, days*^b^*	Body weight, mg*^b^*	Pulse-pair rate, msec*^b^*	Peak amplitude*^b^*	Asynchrony interval, μsec*^b^*
FL-BC	2007	7	119	41.5 (3.67)	15.2 (2.31)	73.6 (6.37)	72.1 (14.39)	666.9 (353.75)
		8	117	41.8 (3.90)	15.1 (2.37)	73.7 (6.45)	67.9 (14.33)	650.1 (347.15)
	2008	1	133	37.9 (2.32)	15.7 (2.10)	73.9 (6.84)	67.8 (14.31)	767.4 (433.36)
		2	46	40.5 (3.76)	15.6 (2.53)	74.9 (5.93)	70.3 (11.36)	670.0 (310.96)
		3	41	38.0 (2.79)	15.1 (2.05)	74.7 (5.69)	67.6 (12.00)	679.3 (394.81)
KS-BC	2007	1	80	41.0 (1.21)	15.1 (1.64)	75.2 (5.44)	79.9 (15.29)	729.5 (351.30)
		2	83	42.7 (4.32)	14.1 (1.94)	75.5 (6.80)	76.0 (17.64)	750.8 (359.20)
		3	74	43.8 (5.28)	13.9 (2.02)	74.8 (6.71)	72.7 (14.16)	775.1 (408.93)
	2008	4	78	44.9 (5.98)	11.7 (1.52)	74.9 (5.22)	66.6 (13.36)	717.6 (354.51)
		5	99	43.3 (4.23)	12.0 (1.76)	75.1 (5.56)	66.5 (13.16)	756.3 (411.32)
		6	31	42.6 (3.26)	11.5 (1.42)	75.2 (5.97)	58.5 (8.24)	768.9 (324.56)
KS-SG	2007	4	78	43.8 (3.06)	14.1 (2.01)	75.8 (4.87)	77.4 (18.20)	709.6 (337.68)
		5	67	40.4 (1.05)	15.4 (1.80)	75.3 (5.73)	76.3 (14.42)	680.2 (341.25)
		6	53	40.6 (0.77)	15.5 (2.98)	76.0 (6.30)	78.3 (16.52)	652.6 (354.05)

a Experimental individuals were derived from a series of families (each derived from a single F_1_ intercross animal) generated across two years.

b Mean (standard deviation) for each phenotype in each family. Peak amplitude is relative, unit-less measure (Limousin and Greenfield 2009; Gleason *et al.* 2016).

We examined correlations among all quantitative traits in each mapping population, as this can show how variation of one trait influences that of another, and potentially also indicates genetic correlations among traits. The pattern of correlations among pairs of traits was similar across the three mapping panels (Table 2), suggesting phenotypic associations are broadly maintained. Development time and body weight were significantly negatively correlated in all three populations (Table 2), a correlation previously reported by Gleason *et al.* (2016), implying longer development time results in lower body weight. Both backcross populations, and to some extent the segregant population, show a positive correlation between peak amplitude and body weight (Table 2). This correlation was already noted in several previous studies in the *A. grisella* system (Jang and Greenfield 1998; Brandt and Greenfield 2004; Alem *et al.* 2013; Gleason *et al.* 2016), and suggests larger males are able to emit songs with a higher peak amplitude. Body weight also shows a weak negative correlation with pulse-pair rate in all three populations (Table 2), as previously shown by Brandt and Greenfield (2004), implying larger males generate songs with lower pulse-pair rates on average. The effects of body weight suggest that at least some of the variation we see in song structure in our mapping panels is due to variation in body size.

**Table 2:** Correlations among phenotypes within each population

Popn	Phenotype	Development time	Body weight	Pulse-pair rate	Peak amplitude
FL-BC	Body weight	−0.240 ****			
	Pulse-pair rate	0.017 ^ns^	−0.095 **		
	Peak amplitude	−0.112 **	0.287 ****	−0.112 **	
	Asynchrony interval	−0.076 ^ns^	0.067 ^ns^	−0.027 ^ns^	0.027 ^ns^
KS-BC	Body weight	−0.216 ****			
	Pulse-pair rate	−0.012 ^ns^	−0.081 *		
	Peak amplitude	−0.163 ***	0.522 ****	−0.056 ^ns^	
	Asynchrony interval	0.057 ^ns^	0.048 ^ns^	−0.083 *	0.062 ^ns^
KS-SG	Body weight	−0.386 ****			
	Pulse-pair rate	0.060 ^ns^	−0.168 **		
	Peak amplitude	−0.110 ^ns^	0.118 *	−0.093 ^ns^	
	Asynchrony interval	−0.049 ^ns^	0.044 ^ns^	−0.005 ^ns^	−0.039 ^ns^

Significance values for Pearson correlation coefficients are: ^ns^, not significant at the 10% level; *, *P* < 0.1; **, *P* < 0.05; ***, *P* < 0.001; ****, *P* < 0.00001.

We found just two, relatively weak correlations among the three song traits (Table 2); a negative correlation between pulse-pair rate and peak amplitude in the FL-BC population, and a negative correlation between pulse-pair rate and asynchrony interval in the KS-BC population, the latter previously reported both by Collins *et al.* (1999) and by Gleason *et al.* (2016). The relationships among these traits have the same sign in the other populations, but are not significant. Thus, any genetic association between song traits is at best extremely subtle, and these traits are most likely impacted by independent genetic and environmental factors.

### Placing markers on linkage groups

Previous attempts to genetically dissect phenotypic variation in the *A. grisella* system have employed AFLP-based maps (Limousin *et al.* 2012; Alem *et al.* 2013) or relatively few markers that collectively have not tagged the full complement of chromosomes in the system (Gleason *et al.* 2016). To mark all linkage groups with sequence-based markers we used a genotyping-by-sequencing approach to generate thousands of markers discriminating the KS and FL parental strains, and then used Lep-MAP2 (Rastas *et al.* 2013, 2016) to place 5,721-12,801 markers on 30 linkage groups in each population (Table S3). Thirty linkage groups is consistent with the haploid number of chromosomes observed through karyotyping by Limousin *et al.* (2012).

### Associating linkage groups with chromosomes from other sequenced lepidopterans

To facilitate future exploration of the *A. grisella* genome in the context of other lepidopterans, we tied our linkage groups to the chromosomes of *Heliconius melpomene* (Heliconius Genome Consortium 2012) and *Bombyx mori* (Xia *et al.* 2004) by leveraging our draft genome assembly (for full details of the assembly see below). First, we used BLAST to place marker sequences on genome scaffolds, thereby tying scaffolds to linkage groups. Subsequently, and again using BLAST, we associated those linkage group-associated *A. grisella* genome scaffolds with *H. melpomene* and *B. mori*. This resulted in connecting 24/30 of our linkage groups to *H. melpomene* chromosomes and 20/30 to *B. mori* chromosomes (Table S4). Our results are consistent with the previously reported homology between the chromosomes of *H. melpomene* and *B. mori* (Heliconius Genome Consortium 2012).

To confirm that this analysis correctly identified the *A. grisella* Z chromosome, we extracted protein sequences from known Z-linked genes from *B. mori* and *Melitaea cinxia* (Ahola *et al.* 2014), and used BLAST to associate them with our draft genome. We found that 206/654 *B. mori* proteins aligned to 149 *A. grisella* scaffolds, and 141/572 *M. cinxia* proteins aligned to 116 *A. grisella* scaffolds. Seventy-three of these scaffolds are in common, strongly suggesting they reside on the Z in *A. grisella*. Indeed, we associated 48/73 of these scaffolds to linkage groups (see below), and 43/48 are placed on the Z chromosome.

Our use of a population of segregants allowed us to find markers linked to the Z chromosome, and support these homology-based analyses. All males from the KS-SG population had one, intact Kansas-derived Z chromosome and one, intact Florida-derived Z chromosome. The specific pattern of inheritance of Z-linked markers in this population initially, and incorrectly appeared as severe segregation distortion during the Lep-MAP2 linkage group assignment, but ultimately this property allowed us to confirm that such markers were on the Z.

### Difficulty ordering markers within linkage groups

**Figure 1:**
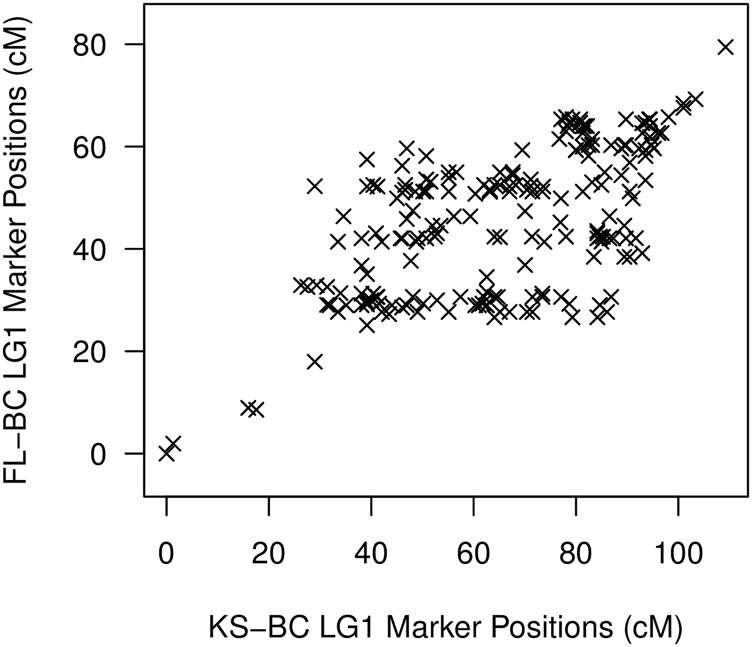
Marker order is not preserved between backcross populations. The genetic positions (in cM) of all markers on linkage group 1 (LG1) that are shared between the FL-BC and KS-BC populations. If relative marker position was consistent between backcrosses, points would appear on a line/curve.

**Figure 2:**
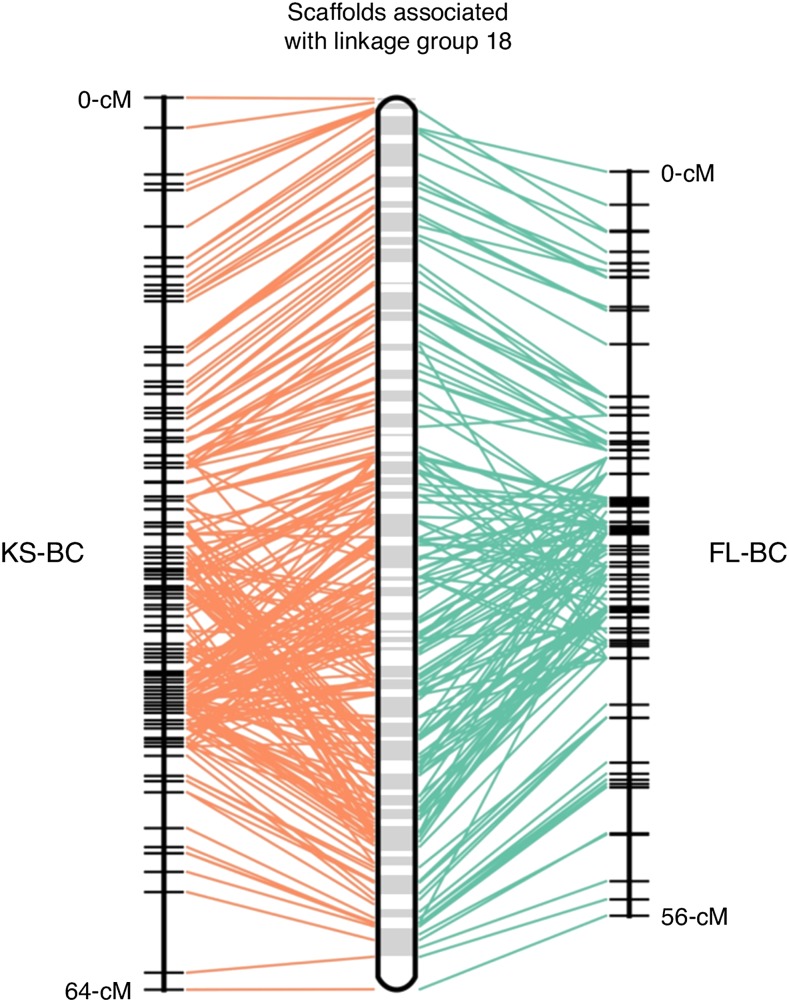
Marker order inconsistency between backcross populations. ALLMAPS was used to map genetic markers to our genome scaffolds separately for each backcross. Blocks in the central chromosome represent scaffolds, and lines connect these physical positions to the genetic positions in each map for markers on linkage group 18. The presence of crossed lines indicates map order inconsistency, which is particularly evident in the central portion of the chromosome.

**Figure 3:**
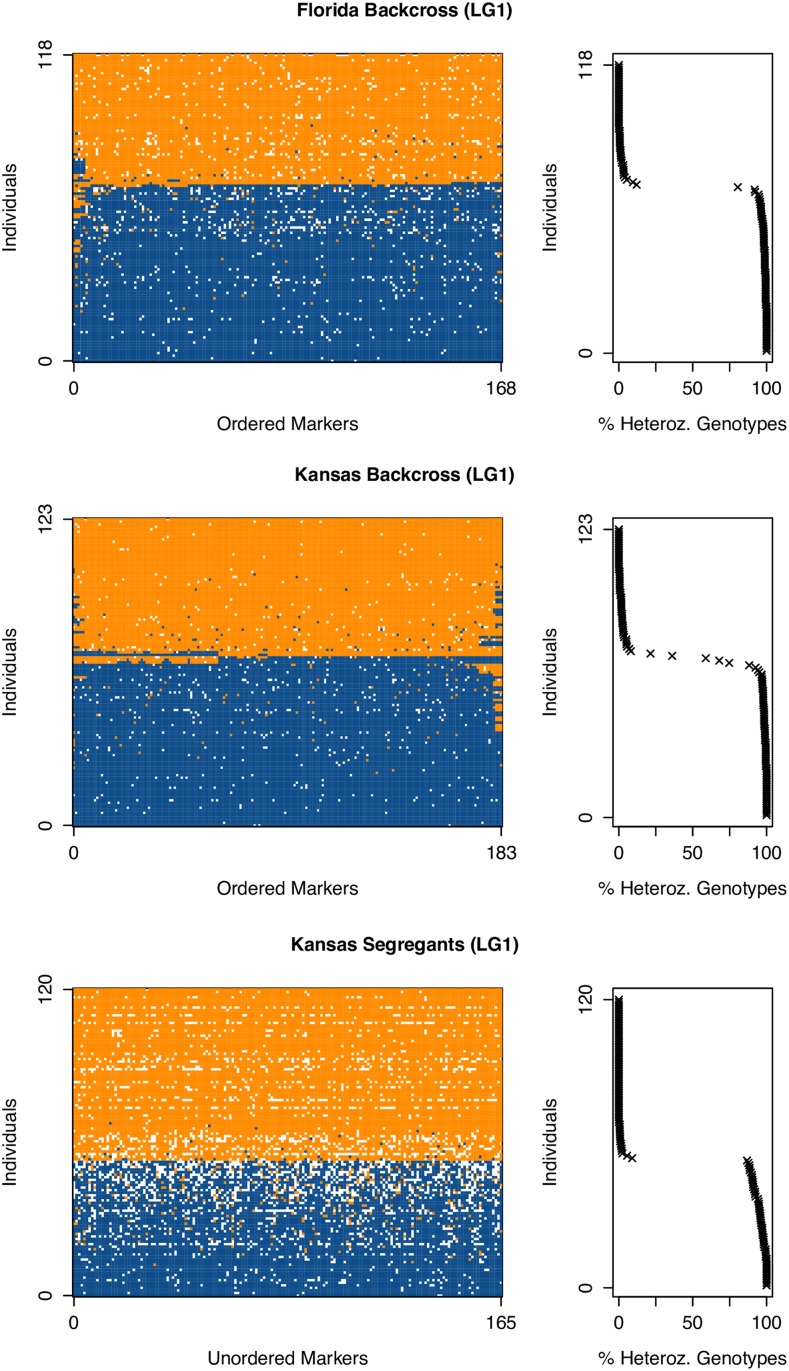
Limited numbers of crossovers in backcross populations. The panels on the left show genotypes for individuals (rows) and markers (columns) for each mapping population for linkage group 1 (LG1). Heterozygous genotypes (FL/KS) are shown in blue, homozygous genotypes (either FL/FL or FL/KS) are shown in orange, and no-calls are shown in white. Only a subset of individuals and markers are presented in the figure to minimize the number of no-call genotypes shown. In the recombinant, backcross populations, with few exceptions (generally near the ends of the linkage groups), the majority of individuals exhibit the same genotype call for the entire chromosome. This is particular clear in the panels on the right, which show the fraction of called genotypes in each individual that are heterozygous.

In examining marker order between the genetic maps derived from the two backcross populations (FL-BC and KS-BC) we found considerable inconsistency. While markers at the termini of each linkage group were largely consistent in order between the two backcrosses, markers in the middle of each linkage group were scrambled. Figure 1 highlights this phenomenon for linkage group 1. If marker order were preserved between backcross populations, markers would fall along a line. We observed the same pattern when using the software ALLMAPS (Tang *et al.* 2015), which allowed us to map genetic markers to the set of physical *A. grisella* scaffolds from our genome assembly. ALLMAPS showed that markers common to both backcross populations always mapped to the same scaffold, but that the marker order defined by each backcross-specific genetic map was distinct (Figure 2 demonstrates this pattern for linkage group 18). To explore this pattern further we plotted genotypes for all individuals in a population at all markers, in the order defined by the genetic map. As exemplified by linkage group 1 (Figure 3), crossovers appear to be relatively rare in the pair of backcross populations, but as expected are absent in the segregant population. Most backcross individuals are *either* homozygous *or* heterozygous for an entire linkage group, and when crossovers are evident they are near the ends of the linkage groups. The apparent scarcity of crossover events provided minimal information to Lep-MAP2 to assist with marker ordering, and is likely why markers in the middle of the linkage groups are inconsistently-ordered between backcross populations (Figures 1 and 2).

Genotyping error could also lead to a spurious reduction in crossover rate and incorrect marker order, and we evaluated the level of genotyping error in our dataset using two strategies. First, we took advantage of the fact that Gleason *et al.* (2016) used a set of KS-BC individuals that overlapped with the set we employed here, but genotyped a different series of markers with an entirely different technology. We compared genotypes between the two studies at markers jointly mapped to the same genome scaffold via BLAST. Across 32 positions, and considering 161-359 individuals per position, the mean percentage of identical genotypes was 96.9%, with just 1/32 having a percent identity below 94% (Table S5). Thus, the marker genotype data we employ in this study appears to be accurate.

Second, we examined the error rate of our genotyping pipeline using the KS-SG segregant population, as here the genotypes of all markers on a given chromosome from a given individual should be identical. Indeed, this is the pattern we observe, with all genotypes on an individual chromosome either being typically homozygous, or typically heterozygous (Figure 3; Figure S2). Nonetheless, genotyping error is not absent from our study. In linkage group 1 of the KS-SG population (Figure 3) a greater number of spurious “homozygous” (orange) calls occur in individuals with majority heterozygous (blue) chromosomes than the reverse. Additionally, when we consider only those chromosomes from KS-SG where at least 30% of markers receive a genotype call, the average frequency of heterozygous calls on the Z is 93.8% (given the cross design, all KS-SG Z-linked loci should be heterozygous), on majority heterozygous autosomes is 93.2%, and on majority homozygous autosomes is 0.01%. These observations imply that our genotyping pipeline undercalls heterozygotes. This is a common issue when applying genotyping-by-sequencing technologies to non-inbred diploid organisms such as our backcross individuals, because a true heterozygote may appear as a homozygote if the alternative allele is simply not sampled by a read, whereas for a true homozygote to appear as a heterozygote a specific sequencing error must occur.

Our use of a fairly common-cutting restriction enzyme (*Ase*I, AT^TAAT) likely contributed to our biased genotyping error, since it resulted in a very large number of markers, each of which we covered only shallowly with sequencing reads. To test whether greater numbers of crossover events, and enhanced consistency in marker order between backcrosses, could be achieved using a dataset with a reduced potential for genotyping error, we repeated the genotype calling step in all three populations, but increased the read depth requirement from 5 to 8 (using *genotypes* in Stacks, see “Materials and Methods”). This new set of genotype calls yielded a similar pattern of apparently rare crossovers and backcross-to-backcross marker order inconsistency.

Overall, it does not appear that genotyping error has dramatically affected reconstruction of the haplotypes in our experimental individuals. Instead, it appears that our *A. grisella* backcross mapping populations are subject to relatively low effective levels of intrachromosomal recombination over the bulk of the physical length of each chromosome. This observation could be due to crossing over events occurring normally, but being localized nearly exclusively to the very ends of chromosomes. Alternatively, our population could be subject to a very low rate of recombination initiation, such that very few Holliday junctions are formed, or the events that do occur could be preferentially resolved into non-crossover molecules. Regardless of the mechanism, the relatively short *A. grisella* genetic map in the present study is supported by the map generated by Gleason *et al.* (2016), who genotyped 75 SNP markers in a set of individuals that encompasses the KS-BC population we employ here. Their map yielded 20 linkage groups, 17 of which had lengths below 20-cM. The AFLP-based mapping study of Alem *et al.* (2013) also yielded a short map, with linkage groups of 12-66 cM in length. These studies suggest that laboratory intercross populations of *A. grisella*, or potentially *A. grisella* as a species, generate relatively few crossovers in each meiosis that provide utility for genetic mapping. However, further study would be required to establish the generality of our observation, and understand the biological basis of the phenomenon.

### Markers for QTL mapping

The modest number of informative crossover events per linkage group makes it challenging to identify QTL containing limited collections of genes. Furthermore, given we cannot be confident of genetic marker order across the bulk of each chromosome, any sub-chromosomal positions may be incorrect. Thus, we collapsed all genotyping data for each individual for each linkage group to a single, consensus genotype (see “Materials and Methods”). This approach has the advantage that it yields a much smaller number of markers to be tested in QTL mapping (*i.e.*, 30), reducing the multiple testing burden over a mapping design with hundreds to thousands of markers. Additionally, in the event that all causative loci on a linkage group act in the same direction (*e.g.*, all KS-derived alleles increase phenotype), testing for associations between phenotypes and entire chromosomes increases power to detect effects, since the effects of multiple, small-effect QTL on a chromosome are aggregated. Of course, since our approach relies on the net effect of a chromosome being different from zero, chromosomes harboring loci of equal and opposite effect on phenotype will not be detected.

A clear drawback of our approach is that we cannot map QTL to sub-chromosomal positions, although we contend that the recombination landscape of our *A. grisella* populations does not readily allow this. Nonetheless, considering the number of linkage groups (N = 30) and the number of genes in our genome annotation (estimated to be 15,848 - see below), mapping a QTL to a chromosome resolves to a few hundred genes. This is approximately the same resolution achievable in an equivalent backcross or F_2_ QTL study in the elite *Drosophila melanogaster* model system (Mackay 2001).

### QTL mapping results

Using marker regression, and accounting for variation due to family by including a covariate during analysis, we mapped variation for our five phenotypes to chromosomes in each of the three mapping panels. Additionally, given the significant correlations between body weight and both pulse-pair rate and peak amplitude (Table 2), we also attempted to map QTL for these song traits after correcting for body weight variation (see “Materials and Methods”). We set genomewide LOD thresholds for significance via permutation testing (Churchill and Doerge 1994), employing three thresholds; α=0.05, the generally-accepted significance level for detection of QTL, and additionally α=0.1 and α=0.2 that allowed us to explore weaker QTL effects.

We identified a number of strongly-supported and suggestive chromosomal effects for the three male song traits, pulse-pair rate, peak amplitude, and asynchrony interval, and the two life history traits, development time and body weight (Table 3). Considering only those effects surviving the most stringent level of statistical significance (α=0.05) we identified two QTL for pulse-pair rate on linkage groups 11 and 13 in KS-BC (both of which are retained following correction for body weight, Table S6), one for asynchrony interval on linkage group 20 in KS-BC, one for development time on linkage group 7 in KS-BC, one for body weight on linkage group 12 in KS-BC, and an additional body weight QTL on linkage group 28 in KS-SG. The effects of these QTL are all fairly modest, each explaining 2.37–8.68% of the phenotypic variance in the mapping population (Table 3), with the largest effect seen for the development time QTL in KS-BC. Similarly low effect sizes were estimated at QTL mapped in Gleason *et al.* (2016). Given the relatively low mapping power we have to identify small-effect loci (Table 3), and since our target traits show significant heritability (Collins *et al.* 1999; Brandt and Greenfield 2004), our results suggest all traits measured are highly polygenic, with genetic contributions to phenotype from an array of variants, many with very small effects on phenotype.

**Table 3:** Summary of mapped QTL

Popn	Phenotype	Linkage group	LOD	Threshold (α)	Variance Expl (%)*^d^*	Effect*^e^*	Power*^f^*
FL-BC	Development time	14	1.84	0.1	1.08	−1.20	0.38
	Body weight	8	2.14	0.1	1.92	−0.95	0.80
	Pulse-pair rate	15*^a^*	1.86	0.1	1.55	2.68	0.79
	Peak amplitude	4*^a^*	1.74	0.2	2.38	6.92	0.96
		5*^a^*	1.67	0.2	2.13	6.41	0.91
KS-BC	Development time	7*^b^*	8.71	0.05	8.68	−3.84	1.00
	Body weight	7*^b^*	1.96	0.1	0.27	0.33	0.02
		12	2.33	0.05	3.53	1.17	0.98
	Pulse-pair rate	11*^c^*	2.66	0.05	2.83	−2.78	0.90
		13*^c^*	2.18	0.05	2.41	−2.82	0.91
	Asynchrony interval	20	2.16	0.05	2.37	167.52	0.86
		22	1.70	0.2	1.66	−137.07	0.57
KS-SG	Development time	12	1.97	0.1	4.90	1.66	0.81
		19	1.68	0.2	4.74	−1.69	0.83
	Body weight	28	2.60	0.05	7.27	1.82	0.97
	Peak amplitude	11*^c^*	1.85	0.1	4.47	10.03	0.74

a These QTL are not replicated after correcting phenotypes for body weight variation (see Table S6).

b QTL for these phenotypes on these linkage groups were identified previously by Gleason *et al.* (2016).

c These QTL replicate after correcting phenotypes for body weight variation (see Table S6).

d Calculated via the R/qtl *fitqtl* function.

e Calculated via the R/qtl *fitqtl* function. Describes the phenotypic effect of substituting a FL-derived allele for a KS-derived allele.

f The statistical power to detect a QTL of the stated effect using our experimental design (see “Materials and Methods”).

To attempt to uncover other phenotypically-relevant loci, and specifically to identify associations with other traits at those QTL positions that survive a more rigorous genomewide threshold, we employed less stringent levels of statistical significance (α=0.1, α=0.2). In the KS-BC population we identified a suggestive QTL for body weight on linkage group 7 that co-localized with the large development time QTL (Table 3). Gleason *et al.* (2016) also identified this pair of QTL (see Table S7 for a list of the QTL mapped in this prior study, and their relationship to peaks identified in the current work). We also saw a suggestive QTL for development time in KS-SG on the same chromosome (linkage group 12) that harbors a body weight QTL in KS-BC. These pair of overlapping QTL may reflect the strong correlation between these two life history traits (Table 2), and suggest either the presence of a locus controlling variation in both traits, or closely-linked loci with distinct effects on each trait. The only other evidence for overlapping QTL was the presence of a weak QTL for peak amplitude on linkage group 11 in the KS-SG population (maintained following body weight correction, Table S6), coincident with a QTL for pulse-pair rate in KS-BC (Table 3). Given the minimal evidence for phenotypic correlation between these song traits (Table 2), the positional overlap is most likely due to chance, and not due to any similarity in the genetic control of trait variation.

Aside from these three instances of across-trait or across-mapping population QTL overlap, even when considering suggestive QTL, no other pairs of QTL map to the same chromosome. Alem *et al.* (2013) similarly observed limited QTL overlap at the chromosomal level in a previous study that examined the same male song and life history traits in an independent *A. grisella* mapping population. There are several explanations for this lack of co-localization. First, some traits may be under independent genetic control, and overlap would not be expected. Indeed, Table 2 shows that variation for many of our traits is not strongly correlated in our mapping panels. Second, even when the phenotypic correlation of two traits is high, the QTL we map have modest effects (Table 3). Our power to detect such QTL was often low (Table 3), limiting our ability to detect the same effect in more than one population. Third, like any QTL study we are subject to the Beavis effect (Beavis *et al.* 1991; Beavis 1994), the phenomenon that the percentage of phenotypic variance explained by QTL that reach a specified level of statistical significance will be overestimated, particularly if the study has a modest sample size. (Other studies have referred to this property as the “winner’s curse”, *e.g.*, Zollner and Pritchard 2007). This suggests that even our small estimated QTL effects are larger than the true effects, compounding the difficulty resolving overlapping pairs of QTL. Fourth, dominance may be a barrier to identifying QTL across reciprocal backcross populations. For instance, even a large effect, but dominantly-acting QTL mapped in the KS-BC population is expected to be invisible in the FL-BC population. Finally, an important technical concern with our chromosome-by-chromosome mapping approach is that if more than one causative locus is present on a chromosome, and the direction of the individual effects sums close to zero, a single consensus chromosome test will reveal no effect. Only much larger mapping panels that incorporate much greater numbers of crossover events, perhaps generated by some kind of advanced intercross design (Darvasi and Soller 1995), are likely to be successful at identifying small effect loci impacting behavioral and morphological variation in *A. grisella*.

### Comparison to previous QTL maps

We compared our QTL results with other studies mapping the same traits in *A. grisella*. Two were based on genetic maps derived solely from anonymous AFLP markers (Limousin *et al.* 2012; Alem *et al.* 2013), so we were unable to resolve chromosome homology among studies, making chromosome-by-chromosome comparison impossible. We were able to directly compare our data to Gleason *et al.* (2016) who used gene-based markers, so we could establish which of their markers map to our *de novo* assembled scaffolds, and translate among their and our linkage group identifiers (Table S2).

Gleason *et al.* (2016) identified eight QTL in a Kansas backcross mapping population, of which our KS-BC individuals were a subset. Just two QTL were apparently identified in both studies (Table 3, Table S7); our development time QTL on linkage group 7 (also our largest-effect QTL), and our suggestive QTL for body weight also on linkage group 7 overlapped QTL for the same traits in Gleason *et al.* (2016). The other six QTL identified by Gleason *et al.* (2016) are not recapitulated in the current study. In two cases - QTL for body weight and pulse rate - this may be because the linkage group these QTL are mapped to in Gleason *et al.* (2016) is split into two linkage groups in our genetic map (Table S2, Table S7). A possibility is that these QTL are spurious, and were generated by incorrectly joining linkage groups due to limited marker density in the previous study. The remaining four QTL identified by Gleason *et al.* (2016), and not identified here, all have small effects (Table S7) and may not have been found simply due to power deficits, particularly because the number of KS-BC individuals we employed was slightly lower than used by Gleason *et al.* (2016). In addition, the two studies used radically different marker sets (75 SNPs *vs.* thousands of markers), and employed very different analytical methodologies; Gleason *et al.* (2016) used composite interval mapping (Zeng 1993, 1994), whereas we used chromosome-by-chromosome marker regression. Such technical differences could easily explain the differences in result, particularly if our traits are highly polygenic, because the identification of small-effect functional loci might be particularly sensitive to the precise mapping strategy applied.

Similar methodological and power concerns might explain why we were able to identify novel QTL (Table 3) not identified by Gleason *et al.* (2016). In addition, we also examined the reciprocal backcross (FL-BC), so if any loci we map segregate for dominantly-acting alleles in the cross, the ability to find such variants in reciprocal backcross populations will differ.

### Genome assembly and annotation

A goal of any mapping project is to enable the identification of genes contributing to trait variation. Hence, we assembled a draft genome of *A. grisella* (Table 4) using both short- and long-insert sequencing libraries and short-read (100-bp) Illumina sequencing. The total, end-to-end length of the assembled scaffolds is 418-Mb, half of the bases are in scaffolds 87.3-Kb or longer, and the assembly has a GC content of 32.4%, which is on par with other sequenced lepidopteran genomes (*e.g.*, Zhan *et al.* 2011). To assess completeness of the set of scaffolds we used CEGMA (Parra *et al.* 2007, 2009), and showed that our assembled scaffolds contain 196/248 (79.03%) intact core eukaryotic genes. Investigators could likely obtain a more contiguous, and more complete assembly by adding long-read, single molecule sequencing data to our short-read sequencing dataset in the future (Chakraborty *et al.* 2018; Baldwin-Brown *et al.* 2018).

**Table 4:** Genome assembly statistics

Number of scaffolds	All	74,159
	>1-Kbp	12,067 (16.3%)
	>10-Kbp	6,202 (8.4%)
	>100-Kbp	1,117 (1.5%)
Scaffold length (bp)	N50*^a^*	87,338
	Summed*^b^*	418,422,425
	Longest	731,388
	Mean	5,642
	Median	185
Fraction of N bases in assembly (%)		2.22
GC content of assembly (%)		32.4

a Half of the bases in the assembly are in scaffolds at least this long.

b The summed, end-to-end length of all scaffolds.

To localize scaffolds to linkage groups we mapped our set of genetic markers to the genome assembly using BLAST, tying 3,099 scaffolds to linkage groups using this approach. While this represents a small minority of the total number of assembled scaffolds (4.2%, Table 4), the scaffolds linked to linkage groups comprise 63.1% of the total length of the assembly. Thus, our genetic markers placed the majority of long scaffolds onto linkage groups. Unfortunately, given our inability to confidently generate an ordered genetic map due to the apparent lack of crossovers in our mapping population, we cannot accurately order or orient scaffolds within linkage groups. If very long scaffolds could be produced in the future via single molecule sequencing, it would be straightforward to physically order markers along linkage groups, better connect the physical and genetic maps, and perhaps increase the resolution of the QTL maps we were able to produce. To facilitate such work we have released all raw and processed genome data associated with this study (see “Data availability”).

To annotate scaffolds we used MAKER2 (Holt and Yandell 2011), identifying 15,848 predicted genes. The number of genes we found is not dissimilar to the 12,669 predicted protein-coding genes identified in *H. melpomene* (Heliconius Genome Consortium 2012) or the 12,901 identified in *B. mori* (Xia *et al.* 2004). MAKER2 scores annotated gene models on a 0-1 Annotation Edit Distance (AED) scale (Eilbeck *et al.* 2009), where lower values indicate better agreement between the annotation and the supporting evidence. The AED scale provides a useful statistic for understanding the quality of an annotation (Holt and Yandell 2011). We found that 91.5% of the predicted genes in our *A. grisella* draft genome have AED values less than or equal to 0.5, suggesting that a large fraction were accurate. Using BUSCO (Simão *et al.* 2015) we sought to identify known single-copy, arthropod genes (see Waterhouse *et al.* 2013) among our annotated gene set. BUSCO identified 80.7% (860/1066) of such genes as complete, with all but 18 of the 860 being single copy in our annotation. A further 12.6% (134/1066) of the test genes were present in our annotation, but fragmented, while 6.8% (72/1066) were missing entirely. Thus, our annotation pipeline has likely identified the bulk of the protein-coding genes in *A. grisella*.

Nearly two thousand of the genes were assigned a predicted function based on sequence similarity with a gene annotated in a related organism, including an array of conserved enzyme genes, genes encoding subunits of the basal transcription machinery, genes for cuticular proteins, detoxification cascade components (*e.g.*, cytochrome P450s), odor and gustatory receptors, and so on. Yet despite this sophisticated genome annotation, most of the genes in the *A. grisella* draft genome were not associated with any predicted function. Notably, even in elite model genetic systems such as *D. melanogaster*, subjected to significant gene-by-gene and genomewide functional exploration, a significant fraction of known genes still have only a basic annotation. The functional annotation of the *A. grisella* draft genome we have constructed would be enhanced by a more detailed comparison of the predicted gene sequences with those from the array of related, lepidopteran and insect genomes that have now been sequenced, and the data we provide should facilitate such comparisons. However, even then there will be no substitute for detailed, functional gene characterization directly within the *A. grisella* system, using both genome-scale technologies (*e.g.*, RNAseq, ATACseq), and gene-specific functional tools (*e.g.*, CRISPR/Cas9 editing which has been successful in lepidopteran systems, see Zhang *et al.* 2017).

By virtue of linking scaffolds to linkage groups we were able to associate between 100 and 576 genes with each linkage group (Table S8), although just over five thousand genes are resident on scaffolds that could not be placed on linkage groups using the present set of markers. While we have only succeeded in elucidating a fraction of the genes on each linkage group, these will ultimately assist with associating genes to the loci mapped for male song and life history traits in this, and in future studies in the *A. grisella* system.

### Concluding thoughts

Our study brought the strengths of next-generation sequencing technologies to a non-model insect species, *Achroia grisella*, to better characterize the genome of the organism, improve the genetic and genomic resources available to the community, and build upon previous work dissecting the genetic basis of a sexually-selected behavioral phenotype exhibited by males of the species. We were able to assemble an accurate, although fragmented, draft genome of *A. grisella* that has an N50 length of >87Kb. Following annotation of the assembled scaffolds we identified nearly 16,000 genes, which evidence suggests represent the bulk of the protein-coding genes of the organism. By virtue of generating a large number of progeny from a cross between a pair of inbred lines, and genotyping these animals for a genomewide set of sequence-based markers, we were also able to assign hundreds of markers to each of the 30 linkage groups harbored by *A. grisella*. In turn, the high marker density allowed us to assign >3,000 long scaffolds to the linkage groups, and associate >63% of the total length of the *de novo* assembly, and >10,000 annotated genes with chromosomes. To facilitate future exploration by investigators we have tied our linkage groups to chromosomes from the sequenced lepidopterans *H. melpomene* and *B. mori*, and have made all our data publicly available.

The principal difficulty we faced, which made it challenging to produce a high-resolution genetic linkage map, order and orient scaffolds along the length of chromosomes, and carry out QTL mapping at sub-chromosomal resolution, was the surprisingly low frequency of crossovers we observed in our data. Multiple lines of evidence indicated this observation was due to rare intra-chromosomal recombination in individuals from our mapping population, and was not the result of inaccurate genotype data. While previous genetic mapping studies in *A. grisella* also indicate a relatively short map length, indicative of low crossover frequency, given the modest sample of genotypes interrogated by laboratory mapping studies, we cannot be confident our results highlight a species-wide phenomenon. Using the resources we outline in the present study future investigations could examine crossover frequency in backcross or F_2_ populations derived from an array of *A. grisella* strains, or perhaps directly examine recombination rate in outbred, wild-caught individuals. If low effective crossover rates are a feature of the species, then whatever the mechanistic basis behind this phenomenon, genetic dissection of male song in *A. grisella* will continue to be challenging. Advanced generation recombinant mapping populations will need to be established by multiple generations of interbreeding in order to produce a mapping population harboring larger numbers of recombination breakpoints throughout the physical length of the genome. To take advantage of these additional crossovers, long-read, single molecule sequencing would additionally be desirable. Such data would lead to a more contiguous genome, would allow markers to be physically ordered along chromosomes, and would allow true interval QTL mapping techniques to be employed.
